# Meta-Analysis in Genome-Wide Association Datasets: Strategies and Application in Parkinson Disease

**DOI:** 10.1371/journal.pone.0000196

**Published:** 2007-02-07

**Authors:** Evangelos Evangelou, Demetrius M. Maraganore, John P.A. Ioannidis

**Affiliations:** 1 Clinical and Molecular Epidemiology Unit, Department of Hygiene and Epidemiology, University of Ioannina School of Medicine, Ioannina, Greece; 2 Department of Neurology, Mayo Clinic College of Medicine, Rochester, Minnesota, United States of America; 3 Biomedical Research Institute, Foundation for Research and Technology-Hellas, Ioannina, Greece; 4 Institute for Clinical Research and Health Policy Studies, Tufts-New England Medical Center, Tufts University School of Medicine, Boston, Massachusetts, United States of America; Fred Hutchinson Cancer Research Center, United States of America

## Abstract

**Background:**

Genome-wide association studies hold substantial promise for identifying common genetic variants that regulate susceptibility to complex diseases. However, for the detection of small genetic effects, single studies may be underpowered. Power may be improved by combining genome-wide datasets with meta-analytic techniques.

**Methodology/Principal Findings:**

Both single and two-stage genome-wide data may be combined and there are several possible strategies. In the two-stage framework, we considered the options of (1) enhancement of replication data and (2) enhancement of first-stage data, and then, we also considered (3) joint meta-analyses including all first-stage and second-stage data. These strategies were examined empirically using data from two genome-wide association studies (three datasets) on Parkinson disease. In the three strategies, we derived 12, 5, and 49 single nucleotide polymorphisms that show significant associations at conventional levels of statistical significance. None of these remained significant after conservative adjustment for the number of performed analyses in each strategy. However, some may warrant further consideration: 6 SNPs were identified with at least 2 of the 3 strategies and 3 SNPs [rs1000291 on chromosome 3, rs2241743 on chromosome 4 and rs3018626 on chromosome 11] were identified with all 3 strategies and had no or minimal between-dataset heterogeneity (I^2^ = 0, 0 and 15%, respectively). Analyses were primarily limited by the suboptimal overlap of tested polymorphisms across different datasets (e.g., only 31,192 shared polymorphisms between the two tier 1 datasets).

**Conclusions/Significance:**

Meta-analysis may be used to improve the power and examine the between-dataset heterogeneity of genome-wide association studies. Prospective designs may be most efficient, if they try to maximize the overlap of genotyping platforms and anticipate the combination of data across many genome-wide association studies.

## Introduction

Genome-wide association analyses are increasingly used to identify common genetic variants that determine susceptibility to disease [1], [2]. Several early successes have generated enthusiasm that such hypothesis-free massive-testing methods may succeed [3], whereas many years of candidate gene approaches have yielded limited, and largely irreproducible postulated associations [4]. However, there are still considerable difficulties in discovering common genetic variants of interest. We already have examples where findings initially highlighted by genome-wide approaches have not been replicated by large-scale studies. This situation has arisen for example in Parkinson's disease, where 13 polymorphisms were originally identified as being potentially important for determining the risk of the disease in a two-tier genome-wide association study [5]. Nevertheless, all 13 proposed associations were not replicated by a large-scale effort involving over 12,000 subjects [6]. For most common diseases, the main genetic effects are expected to be small and therefore would require very large studies to capture [7]. Genome-wide association studies published to-date have had mostly modest sample sizes, and even the ongoing efforts sponsored by Wellcome Trust and the GAIN initiative [8], [9] may still be underpowered to detect odds ratios in the range of 1.0–1.3, especially if the genetic variants of interest are not very common.

It is important to maximally exploit the available data from genome-wide association studies and combine information from different such studies performed on the same disease. In the candidate gene era, a very large number of teams independently pursued studies on specific candidate gene variants. While the technical and financial requirements for genome-wide association studies are more demanding, several such studies may still be conducted by independent teams of investigators working on the same disease. This creates a challenge and an opportunity to combine these data with meta-analytic techniques. Meta-analysis has already been accepted as a prime method for examining the consistency, replication, and credibility of proposed genetic associations [10], [11]. However, to our knowledge, no meta-analysis has yet been performed combining data from different genome-wide association studies on the same disease. Here, we have performed such a meta-analysis for Parkinson's disease. We aimed to explore the different meta-analytic strategies that can be pursued and to dissect the limitations that arise in combining such datasets with meta-analytic methods.

## Methods

### Databases

We used publicly available data from two genome-wide association studies of Parkinson's disease (PD). Maraganore et al. [5] used a two-tiered genotyping approach (which will be referred as Mayo tier 1 and Mayo tier 2 for simplicity). In Mayo tier 1, 443 case-unaffected sibling pairs that were discordant for PD were included. Genotyping used the Perlegen platform. For the 205,031 single nucleotide polymorphisms (SNPs) that were polymorphic within the study sample the Hardy-Weinberg equilibrium (HWE) p-value was >0.001 in controls for 198,345 SNPs. The investigators performed a liberalization of the sibling transmission/disequilibrium test (sTDT) to identify SNPs that had significant allele-frequency differences in cases versus unaffected siblings, adjusted for age and sex. For each SNP, odds ratios (ORs), 95% confidence intervals (CIs) and p-values were calculated. There were 1,862 SNPs associated with PD in tier 1 at p<0.01.

In Mayo tier 2a, genotypes and analyses for the 1,862 SNPS selected in tier 1 and for 311 genomic controls were measured in 332 case-unrelated control pairs. Genotyping call rates>80% and HWE p-values>0.001 were achieved for 1,793 SNPs. In Mayo tier 2b, 975 SNPs were selected for further testing with biological or other reasoning (e.g. significant effects on subgroup analyses) regarding susceptibility to PD. Of these, genotyping call rates>80% and HWE p-values>0.001 in controls were achieved for 941 SNPs and ORs, 95% CIs and p-values were calculated. The study is described in more details elsewhere [5]. SNPs, alleles, case and control allele frequencies, ORs, 95% CIs and p-values are available online.

Fung et al. [12] performed a genome-wide association study sponsored by National Institute of Neurological Disorders and Stroke (NINDS) where genotyping was performed on 408,803 unique SNPs combining the Illumina Infinium I and HumanHap300 platforms. The investigators undertook a one-stage genome-wide association study in 276 patients with PD and 276 neurologically normal controls. The samples used for this study were derived from the NINDS Neurogenetics repository hosted by the Coriell Institute for Medical research. For the 408,803 SNPs studied, the genotype call rate was greater that 99% for 395,275 SNPs and greater that 95% for 406,312 SNPs. The HWE p value was >0.001 for 395,493 SNPs. The study is described in more details elsewhere [12]. Raw data are publicly available online at the Coriell Institute website.

### Genetic models and effect sizes

For consistency, all ORs were computed based on the major vs. minor allele contrast, and assignment of minor allele status is based on the allele frequencies of the control samples in the NINDS study.

The Mayo data were originally analyzed using a log-additive model with trend adjusted for age and sex. The OR and 95% CIs from this model were used in order to calculate the natural logarithms of the OR and the standard error of the natural logarithm of the OR for each gene variant. The standard error is given by the difference of the natural logarithms of the upper and lower boundary of the 95% CI, divided by 3.92. Both Mayo tier 1 and tier 2 used matched designs.

The NINDS study examined various types of genetic contrasts including recessive, dominant, and additive models (linear additive, as opposed to log-additive used in the Mayo study) and also provided raw data on alleles for each examined SNP. We calculated the natural logarithm of the allele-based OR and the standard error of the natural logarithm of the OR from the counts of alleles given in cases and controls in the NINDS database. The allele-based OR is practically equivalent to the log-additive model with consideration of trend.

### Meta-analysis: statistical methods

The natural logarithms of the OR estimates were combined to estimate a summary OR using fixed [13] and random effects models[14] using inverse variance calculations. In fixed effects models, the true effect of risk allele is assumed to be the same value in each dataset, whereas in random effects models the risk allele effects for the individual datasets are assumed to vary around some overall average effect. If var(f) is the variance of each effect (here, natural logarithm of odds ratio) in a study and var(r) is the random effects variance, then in fixed effects calculations, each study is weighted by 1/var(f), while in random effects calculations each study is weighted by 1/[var(f)+var(r)]. Therefore, random effects approach is generally considered more conservative, yielding wider confidence intervals. Between-dataset heterogeneity was quantified using the I^2^ metric for inconsistency [15] and its statistical significance was tested with the chi-square distributed Q statistic [16]. I^2^ is provided by the ratio of (Q-df)/Q, where df = the number of degrees of freedom (one less than the number of combined datasets); it is considered large for values above 50% and Q is considered statistically significant for p<0.10 [15], [16]. In the absence of any between-dataset heterogeneity, fixed and random effects estimates coincide.

### Meta-analysis strategies and multiplicity considerations

We considered the following strategies for combining the available datasets. In the two-stage framework, options included enhancement of replication (second-stage) data and enhancement of first-stage data. We also performed joint meta-analyses including all first-stage and second-stage data.

Enhancement of replication data: In this strategy, the Mayo tier 1 are still considered as the first-stage information and the meta-analysis of the Mayo tier 2 and NINDS datasets represent their second-stage independent replication. The number of tested SNPs for which adjustment needs to be made represents those SNPs that have data available in both the Mayo tier 2 and NINDS datasets.

Enhancement of first-stage data: In this strategy, the data from Mayo tier 1 and NINDS datasets were combined by meta-analysis, and a new first-stage with increased power was created. The summary effects derived in the new first-stage that were statistically significant at p<0.05 level (at least by fixed effects) were then examined for replication in the Mayo tier 2 dataset. The number of tested SNPs for which adjustment needs to be made in the enhanced first-stage data is the common SNPs in both Mayo tier 1 and NINDS datasets; in the replication sample (Mayo tier 2), it is those SNPs that have p<0.05 at least by fixed effects in the combined Mayo tier 1 and NINDS datasets and have also been assessed in the Mayo tier 2 dataset.

Joint analysis: In this strategy, we jointly meta-analyzed all three databases (Mayo tier 1, Mayo tier 2, NINDS) to obtain summary effects. This strategy may be applied to all SNPs where data are available in all three datasets; however, it is improper to adjust the results for the number of common SNPs across all three databases, because the selection of SNPs in the Mayo tier 2 dataset is not random. This strategy combines eventually all three databases, but as a first step the Mayo tier 1 and NINDS data are meta-analyzed, and then only those SNPs that still have p<0.05 at least with fixed effects are then considered for inclusion of the Mayo tier 2 data. If x SNPs have p<0.05 based on Mayo tier 1 and NINDS combined, and y of them are also tested in Mayo tier 2, the p-value may be adjusted for the number of SNPs that have data available in both Mayo tier 1 and NINDS multiplied by the fraction x/y. This is approximately correct, if the x SNPs can be considered a random sample of the y SNPs, an assumption which we tested by comparing the distribution of p-values in the x selected versus the y-x non-selected SNPs according to a Wilcoxon rank-sum test (p = 0.34).

All the above corrections for multiplicity of comparisons are conservative and assume that the tested SNPs are independent, while this is an oversimplification. However, they provide a starting point for considering the extent of multiple comparisons involved.

Analyses were performed in STATA 8.2 (College Station, TX). P-values are two-tailed. For SNPs that were selected by more than one strategy and that pertained to a specific gene, we perused Entrez Gene (paying attention to the Process listed in Gene Ontology) and also queried PubMed using the gene name and Parkinson's disease, in order to identify if there is any hint of biological plausibility or evidence relating these genes specifically to Parkinson's disease.

## Results

### Strategy of enhancement of the replication data

A total of 572 SNPs had data available both in the Mayo tier 2 and NINDS datasets (including 45 that were simply used as genomic controls in Mayo tier 2 without being tested at the Mayo tier 1 dataset). Meta-analysis of the Mayo tier 2 and NINDS datasets showed that 38 SNPs were significant at p<0.05 with fixed effects and 26 remained significant at p<0.05 using random effect models (Table 1). Fourteen out of these 26 SNPs were also significant at p<0.01 in Mayo tier 1, but 4 of the significant effects were in the opposite direction compared to the summary ORs obtained by the meta-analysis of the Mayo tier 2 and NINDS datasets; the remaining 10 SNPs may warrant further consideration. There were also 3 SNPs that were used as genomic controls in Mayo tier 2 and 9 SNPs that had not reached the p<0.01 level of significance in Mayo tier 1, but they were included in Mayo tier 2b (biological or other reasoning). Two of these SNPs had genetic effects in the same direction as in tier 1. These 2 SNPs may also warrant further consideration. None of the associations seen in the enhanced replication data would remain formally significant after adjustment for 527 (572–45) comparisons, since the lowest p-value was 0.005.

**Table 1:** Significant summary ORs (p<0.05 at least with fixed effects analyses) and 95% confidence intervals in meta-analysis of Mayo tier 2 and NINDS datasets.

Db SNP ID	Gene name	Fixed effects OR (95% CI)			p-value (unadjusted)	Random effects OR (95% CI)			p-value (unadjusted)	I^2^
Chromosome 1										
rs2038379^a^		1.240	1.027	1.498	0.025	1.243	0.979	1.579	0.074	37.5
** rs2488787** a		**0.824**	**0.694**	**0.978**	**0.027**	**0.824**	**0.694**	**0.978**	**0.027**	0
rs7520966		0.763	0.635	0.916	0.004	0.752	0.512	1.105	0.187	77.7^c^
Chromosome 2										
** rs2372479**	***ABCA12***	**1.292**	**1.093**	**1.527**	**0.003**	**1.292**	**1.093**	**1.527**	**0.003**	0
Chromosome 3										
** rs1000291** a	***FAM79B***	**1.280**	**1.079**	**1.520**	**0.005**	**1.280**	**1.079**	**1.520**	**0.005**	0
** rs1515445** b	***SPATA16***	**1.350**	**1.046**	**1.742**	**0.021**	**1.342**	**1.004**	**1.794**	**0.047**	21.7
** rs7038**		**1.190**	**1.008**	**1.405**	**0.040**	**1.190**	**1.008**	**1.405**	**0.040**	0
Chromosome 4										
** rs1469259** a		**1.290**	**1.016**	**1.639**	**0.037**	**1.290**	**1.016**	**1.639**	**0.037**	0
** rs1836803**		**0.804**	**0.669**	**0.966**	**0.020**	**0.804**	**0.669**	**0.966**	**0.020**	0
** rs2241743** a	***UNC5C***	**1.180**	**1.006**	**1.385**	**0.042**	**1.180**	**1.006**	**1.385**	**0.042**	0
** rs2302565(GC)**		**1.217**	**1.020**	**1.451**	**0.029**	**1.217**	**1.020**	**1.451**	**0.029**	0
rs2313982^a^(M)		1.536	1.141	2.067	0.005	1.528	0.975	2.395	0.065	56.2
Chromosome 5										
** rs969518**	***CPEB4***	**1.202**	**1.018**	**1.419**	**0.030**	**1.202**	**1.018**	**1.419**	**0.030**	0
Chromosome 6										
rs10484586^b^		0.743	0.554	0.997	0.047	0.712	0.424	1.194	0.197	65.9^c^
rs3095352		1.282	1.088	1.512	0.003	1.273	0.938	1.726	0.121	70.7^c^
rs3130653^b^		1.262	1.070	1.490	0.006	1.254	0.899	1.749	0.183	75.2^c^
** rs6910844**		**0.742**	**0.598**	**0.921**	**0.007**	**0.742**	**0.598**	**0.921**	**0.007**	0
rs6929069	*DSP*	1.289	1.000	1.662	0.05	1.302	0.861	1.968	0.211	62.1
** rs9328331** a	***EXOC2***	**0.817**	**0.675**	**0.990**	**0.039**	**0.817**	**0.675**	**0.990**	**0.039**	0
Chromosome 10										
** rs12219199** b	***GPR120***	**1.282**	**1.088**	**1.512**	**0.037**	**1.282**	**1.088**	**1.512**	**0.037**	0
** rs4746308**		**0.739**	**0.567**	**0.964**	**0.026**	**0.739**	**0.567**	**0.964**	**0.026**	0
Chromosome 11										
** rs3018626** a		**1.238**	**1.012**	**1.513**	**0.038**	**1.238**	**1.012**	**1.513**	**0.038**	0
rs368911^a^		0.814	0.685	0.968	0.020	0.833	0.709	0.979	0.064	38.8
** rs485642** a	***MAML2***	**1.202**	**1.000**	**1.444**	**0.050**	**1.202**	**1.000**	**1.444**	**0.050**	0
** rs898309** a		**0.816**	**0.670**	**0.992**	**0.042**	**0.816**	**0.670**	**0.992**	**0.042**	0
** rs9332434(GC)**		**0.833**	**0.709**	**0.979**	**0.027**	**0.833**	**0.709**	**0.979**	**0.027**	0
Chromosome 12										
** rs10492243**		**1.276**	**1.007**	**1.615**	**0.043**	**1.276**	**1.007**	**1.615**	**0.043**	0
** rs342169**	***PPM1***	**1.340**	**1.045**	**1.717**	**0.021**	**1.340**	**1.045**	**1.717**	**0.021**	0
Chromosome 13										
rs2057525^b^	*GPC6*	0.784	0.641	0.959	0.018	0.782	0.567	1.079	0.135	60.9
Chromosome 14										
rs1889720^b^		0.683	0.471	0.991	0.045	0.664	0.333	1.325	0.195	70.7^c^
** rs8020291** b		**1.231**	**1.053**	**1.440**	**0.009**	**1.231**	**1.053**	**1.440**	**0.009**	0
Chromosome 15										
** rs1865997** a		**0.786**	**0.664**	**0.930**	**0.005**	**0.786**	**0.657**	**0.940**	**0.008**	11.5
** rs2460641** a		**1.283**	**1.071**	**1.537**	**0.007**	**1.283**	**1.071**	**1.537**	**0.007**	0
rs613479^b^		1.203	1.016	1.425	0.032	1.201	0.976	1.478	0.084	33.7
rs623941^b^	*CHRM5*	1.266	1.060	1.513	0.009	1.265	0.994	1.609	0.056	45.3
Chromosome 19										
** rs4808631(GC)**		**1.205**	**1.024**	**1.418**	**0.025**	**1.205**	**1.024**	**1.418**	**0.025**	0
Chromosome 20										
** rs6036107**		**1.423**	**1.038**	**1.950**	**0.028**	**1.423**	**1.038**	**1.950**	**0.028**	0
Chromosome X										
** rs5928917** b		**1.192**	**1.026**	**1.386**	**0.022**	**1.192**	**1.026**	**1.386**	**0.022**	0

Associations that are significant (p<0.05) with both fixed and random effects are in bold type.

a Effect in the same direction in Mayo tier 1

b Statistically significant at p<0.01 in Mayo tier 1

c Statistically significant (p<0.10) Cochran's Q

GC: SNP used simply as genomic controls in Mayo tier 2

(M): SNP that was originally proposed to be associated with Parkinson disease risk in the original publication of the Mayo data

### Strategy of enhancement of the first-stage data

The Mayo tier 1 and NINDS datasets shared 32,192 common SNPs. Meta-analyses with fixed effects models for these SNPs, gave 1,503 significant associations at p<0.05. Of these 1,503 SNPs, 173 had been tested also in Mayo tier 2. Eight of these SNPs had also p<0.05 in the Mayo tier 2 dataset and five of them (rs1000291 [chromosome 3], rs2241743 and rs2313982 [chromosome 4], rs3018626 [chromosome 11] and rs2282048 [chromosome13]) had the same direction of effect and they would warrant further consideration based on this strategy (Table 2). Three of the 5 SNPs (rs1000291, rs2241743 and rs3018626) had also been identified with the enhancement of replication data strategy. The lowest p-value in the Mayo tier 2 dataset for an association with the same direction of effect in both stages was 0.0015 (for rs2313982), which would not be formally significant after adjusting for 173 comparisons; this SNP was one that was identified as a candidate SNP in the original Mayo genome-wide association study [5], but was not replicated in the subsequent large-scale replication effort [6]. The SNP rs6050372 in chromosome 20 had a very low p-value (0.000014) in the Mayo tier 1 and NINDS datasets combined, but the observed effect was significant (p = 0.0148) in the opposite direction upon replication in Mayo tier 2.

**Table 2:** Significant associations (at least with fixed effects) from meta-analysis of Mayo tier 1 and NINDS databases that were also considered in Mayo tier 2.

Db SNP ID	Gene	Fixed effects OR (95% CI)	p-value unadjusted	Random effects OR (95% CI)	p-value unadjusted	I^2^	OR (p-value) in Mayo tier 2
Chromosome 1							
rs668556		0.804 (0.671–0.963)	0.018	0.796 (0.606–1.046)	0.101	55.6	1.43 (0.0015)
Chromosome 3							
** rs1000291**	***FAM79B***	**1.348 (1.122–1.620)**	**0.00144**	**1.351 (1.111–1.642)**	**0.003**	**10.9**	**1.32 (0.0237)**
Chromosome 4							
rs2241743	*UNC5C*	1.240 (1.030–1.492)	0.023	1.260 (0.930–1.707)	0.136	62	1.09 (0.034)
rs2313982		1.492 (1.062–2.096)	0.021	1.558 (0.899–2.701)	0.114	59.6	1.21 (0.0015)
Chromosome 11							
rs3018626		1.306 (1.043–1.634)	0.020	1.334 (0.941–1.890)	0.105	56.9	1.34 (0.0365)
Chromosome 13							
rs2282048	*FARP1*	0.822 (0.683–0.990)	0.038	0.806 (0.558–1.166)	0.253	25.3	0.79 (0.0482)
rs8002725		0.744 (0.576–0.961)	0.024	0.734 (0.520–1.036)	0.079	43.5	1.53 (0.0089)
Chromosome 20							
** rs6050372**		**0.644 (0.527–0.786)**	**0.000014**	**0.644 (0.527–0.786)**	**0.000014**	**0**	**1.38 (0.0148)**

Associations that reach p<0.05 in the meta-analysis by both fixed and random effects are in bold type.

### Joint analysis of all three datasets

There were 527 SNPs with data available in all three datasets. Of those, 102 SNPs were statistically significant at p<0.05 using fixed effects and 49 were significant also by random effects (Table 3). The number of statistically significant results seems large, but this is spurious. Mayo tier 2 testing was guided by the Mayo tier 1 data.

**Table 3:** Significant summary ORs (both in fixed and random effects analyses) and 95% confidence intervals computed from meta-analysis of Mayo tier 1, Mayo tier 2 and NINDS datasets.

Db SNP ID	Gene	Fixed effects OR (95% CI)			p-value (unadjusted)	Random effects OR (95% CI)			p-value (unadjusted)	I^2^
Chromosome 1										
rs2038379	*DAB1*	1.324	1.124	1.559	0.00076	1.337	1.078	1.659	0.008	41.5
rs2488787		0.764	0.659	0.887	0.00041	0.756	0.621	0.920	0.005	41.3
rs3748841	*FLJ20972*	1.375	1.118	1.689	0.002	1.383	1.100	1.739	0.006	18.2
rs7520966^a^		0.752	0.644	0.880	0.00036	0.755	0.595	0.957	0.020	56.2
Chromosome 2										
rs1427547		0.795	0.691	0.915	0.00141	0.790	0.666	0.937	0.007	31.6
rs4666255	*ALK*	0.807	0.684	0.952	0.011	0.799	0.652	0.978	0.030	32.1
rs838709	*DGKD*	0.843	0.723	0.983	0.029	0.843	0.723	0.983	0.029	0
Chromosome 3										
rs1000291	*FAM79B*	1.339	1.156	1.551	0.00010	1.339	1.156	1.551	0.00010	0
rs1669215		0.757	0.641	0.894	0.00101	0.754	0.619	0.919	0.005	28.7
rs2243115	*IL12A*	1.324	1.066	1.645	0.011	1.324	1.066	1.645	0.011	0
rs500097^a^	*LOC389142*	0.798	0.684	0.930	0.004	0.777	0.604	1.001	0.050	61.7
rs6445726		1.178	1.027	1.351	0.020	1.183	1.000	1.400	0.049	32.1
Chromosome 4										
rs1469259		1.392	1.131	1.713	0.002	1.392	1.131	1.713	0.002	0
rs2241743	*UNC5C*	1.247	1.084	1.434	0.002	1.250	1.063	1.470	0.007	24.2
rs2313982(M)		1.650	1.270	2.145	0.00018	1.666	1.184	2.346	0.003	39.5
rs6819953		0.715	0.558	0.916	0.008	0.715	0.558	0.916	0.008	0
rs7694392^a^	*BANK1*	1.472	1.158	1.872	0.002	1.627	1.016	2.604	0.043	71.1^c^
Chromosome 5										
rs3213837^a^	*ERBB2IP*	0.813	0.671	0.984	0.033	0.812	0.659	1.000	0.050	16.1
Chromosome 6										
rs1906966		1.201	1.034	1.394	0.016	1.211	1.006	1.457	0.043	33.7
rs9328331	*EXOC2*	0.767	0.648	0.907	0.002	0.767	0.648	0.907	0.002	0
Chromosome 7										
rs10499882^a^	*HMG17P1*	0.812	0.692	0.954	0.011	0.812	0.692	0.954	0.011	0
rs1866571		1.233	1.065	1.427	0.005	1.245	1.028	1.509	0.025	40.7
Chromosome 8										
None										
Chromosome 9										
rs10115467	*LOC648385*	0.828	0.719	0.954	0.009	0.817	0.673	0.992	0.041	45.8
rs3761672^a^		1.202	1.039	1.391	0.013	1.202	1.039	1.391	0.013	0
Chromosome 10										
rs4746308		0.703	0.557	0.887	0.003	0.703	0.557	0.887	0.003	0
rs7079524		0.780	0.646	0.942	0.010	0.770	0.594	0.998	0.048	46.4
Chromosome 11										
rs2282658^a^	*CASP5*	1.253	1.079	1.456	0.003	1.256	1.064	1.483	0.007	18.4
rs3018626		1.320	1.108	1.573	0.002	1.324	1.094	1.602	0.004	14.8
rs368911		0.776	0.669	0.899	0.00075	0.772	0.649	0.918	0.003	27.1
rs485642	*MAML2*	1.288	1.098	1.511	0.002	1.292	1.088	1.534	0.004	12.9
rs651861	*ODZ4*	1.191	1.038	1.366	0.013	1.197	1.023	1.400	0.025	22.3
Chromosome 12										
rs1317852		1.184	1.024	1.368	0.022	1.189	1.003	1.409	0.046	27.5
Chromosome 13										
rs2282048	*FARP1*	0.810	0.701	0.937	0.004	0.805	0.656	0.988	0.038	49.3
rs9316335^a^	*ZDHHC20*	0.851	0.734	0.988	0.034	0.851	0.732	0.990	0.037	3.1
Chromosome 14										
rs175990		1.283	1.088	1.513	0.003	1.297	1.036	1.625	0.024	45.5
rs4280164^a^	*C14orf21*	1.214	1.023	1.439	0.026	1.214	1.023	1.439	0.026	0
Chromosome 15										
rs1865997		0.757	0.653	0.877	0.00021	0.757	0.653	0.877	0.00021	0
Chromosome 16										
rs8047091	*ABCC11*	0.757	0.624	0.919	0.005	0.733	0.542	0.991	0.043	57.3
rs9938490		0.741	0.609	0.901	0.003	0.709	0.507	0.992	0.045	64.6^b^
Chromosome 17										
rs2215290		1.321	1.049	1.664	0.018	1.336	1.015	1.760	0.039	28.5
rs8066468^a^		0.830	0.716	0.961	0.013	0.829	0.714	0.963	0.014	3.3
rs8176318	*BRCA1*	0.815	0.701	0.946	0.007	0.815	0.701	0.946	0.007	0
Chromosome 18										
rs1893963	*DSC2*	0.723	0.557	0.939	0.015	0.717	0.538	0.955	0.023	15.2
Chromosome 19										
rs1363938	*FLJ35784*	1.329	1.022	1.729	0.034	1.330	1.015	1.743	0.038	5.1
rs2387137	*SYT3*	0.812	0.679	0.971	0.023	0.812	0.679	0.971	0.023	0
Chromosome 20										
rs1135961	*PSMA7*	0.784	0.642	0.957	0.017	0.779	0.630	0.965	0.022	11.1
rs6036107		1.473	1.136	1.910	0.003	1.473	1.136	1.910	0.003	0
Chromosome 21										
None										
Chromosome 22										
None										
Chromosome X										
rs5907306		1.167	1.026	1.327	0.019	1.167	1.026	1.327	0.019	0
rs7064448		0.775	0.653	0.920	0.004	0.775	0.653	0.920	0.004	0

a Did not reach p<0.05 by fixed effects in meta-analysis of Mayo tier 1 and NINDS data

b Statistically significant (p<0.10) Cochran's Q for heterogeneity

M: SNP that was originally proposed to be associated with Parkinson disease risk in the original publication of the Mayo data

We limited further the joint analysis to those SNPs where not only data were available in all three datasets, but also had statistically significant results from a first-step meta-analysis of the Mayo tier 1 and NINDS datasets (p<0.05 at least by fixed effects). As stated above in the strategy of enhancement of first-stage data, there were 173 such SNPs. Of those, 72 SNPs were found to be statistically significant at p<0.05 by fixed effects when the three databases were jointly analyzed. Thirty-nine of these SNPs had p<0.05 by random effects models as well (Table 3). None of the associations would remain significant after adjusting p-values by a factor of 32,192×(173/1,503) = 3,705 (see methods). The SNPs with the lowest p-values were rs1000291 and rs1865997 (p = 0.00010 and p = 0.00021 respectively, using random effects calculations). Both SNPs were significant at p<0.05 in the enhancement of replication strategy. Eight more SNPs were among those that warranted further consideration based on the strategy of enhancement of replication data. SNPs rs1000291 had p<0.05 also in the strategy of enhancement of first-stage data.

### SNPs selected in two or more strategies and other evidence

**Figure 1:**
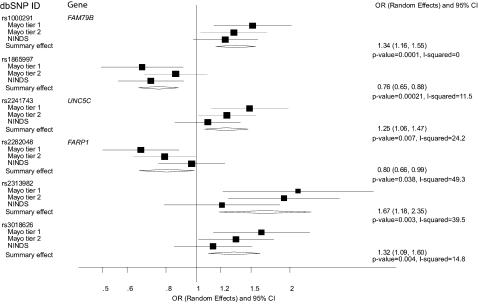
Meta-analyses of the three datasets for the 6 single nucleotide polymorphisms that were selected (p<0.05 unadjusted for multiple comparisons) with at least two of the three strategies. For each polymorphism the forest plot shows the odds ratio and 95% confidence interval for each dataset as well as the summary odds ratio and 95% confidence intervals by random effects calculations. Also shown is the p-value for the summary effect and the I-squared statistic for between-dataset heterogeneity.

Figure 1 shows the results of meta-analyses using the joint analysis approach for 6 SNPs that had p<0.05 according to at least 2 of the three strategies that we employed. Three SNPs (rs1000291 on chromosome 3, rs2241743 on chromosome 4 and rs3018626 on chromosome 11) were selected by all three strategies; the first two had absolutely no between-dataset heterogeneity (I^2^ = 0) and the third had minimal between-dataset heterogeneity (I^2^ = 15%). There was larger, but still not formally statistically significant heterogeneity for the other 3 SNPs that were selected by 2 of the 3 strategies (I^2^ ranging between 24% and 49%). No SNP had p<0.05 and the same direction of effect separately in all 3 datasets.

According to Entrez Gene and PubMed, we found some hints for potential biological plausibility for the *UNC5C* gene where the rs2241743 polymorphism is located. The gene product belongs to the *UNC-5* family of netrin receptors. Netrins are secreted proteins that direct axon extension and cell migration during neural development. *UNC5C* maps to the alpha-synuclein locus of chromosome 4 [17], where the *SCNA* gene is an already well-known Parkinson's disease susceptibility gene. It is tempting to speculate whether the axon guidance pathway may have broader pathogenetic implications for Parkinson's disease, as netrin and netrin receptors have important roles for nigral dopaminergic neurons [18], [19]. Parenthetically, the most significant finding from the study of Maraganore et al. [5] was for a SNP within *SEMA5A*, another axon guidance pathway gene (although that finding has not been independently replicated thus far).

## Discussion

We show using empirical data how meta-analysis can be used to combine information from genome-wide datasets. Meta-analysis is a well-established method to synthesize results and draw conclusions from different studies for a set of related research hypotheses and it has the greater citation impact in the health sciences literature compared to other study designs [20]. When performed appropriately, meta-analysis may enhance the precision of the estimates of the effects of risk alleles, leading to reduced probability of false negative results. The increased availability of information can also lead to rejection of null hypotheses at lower levels of type I error, thus reducing the false discovery rate [21]. In the field of Human Genome Epidemiology, meta-analyses of gene-disease association studies to–date have addressed typically one or a few postulated associations at a time and even large-scale overviews of many meta-analyses have addressed a few dozens of associations at the most [10], [11], [22]. Genome-wide association analyses provide an opportunity to conduct many thousands of SNP-specific meta-analyses concurrently. This may yield some interesting results that are worth pursuing further, as in our datasets. However, the multiplicity of comparisons has to be factored to avoid making exaggerated claims about the promising SNPs that emerge from such meta-analyses. The synthesis and interpretation of gene-disease associations should be cautious, especially when weak associations are considered. Misclassification, confounding (population stratification) and selective reporting may lead to spurious findings [23]. Biological plausibility and other external evidence may be considered as well to interpret the results of the meta-analysis. Here, the identification of a polymorphism is a axon guidance pathway gene is intriguing, but certainly requires independent corroboration and replication before any strong claim can be made.

Our empirical evaluation also revealed several issues that need to be considered in future efforts. First, when different genotyping platforms are used, as in our datasets, the overlap of genetic markers may be suboptimal. The Mayo and NINDS platforms had only modest overlap (only approximately 16% of the Mayo tier 1 dataset SNPs also had data in the NINDS dataset). This is expected to result in large loss of genomic coverage, even if the coverage of each platform is very good [24]. One may consider also juxtaposing and combining data from SNPs that are in very strong linkage disequilibrium or may even consider genic approaches to the data [25].

Second, meta-analyses may lead to spurious or heterogeneous results if the definitions of disease phenotypes and controls are different across the combined datasets. For Parkinson disease, for example, there are many different accepted clinical definitions, but hopefully they do not lead to major discrepancies in diagnosis. Population stratification may also lead to spurious or heterogeneous results in a meta-analysis, if some of the combined studies are affected. In our application, population stratification had been more thoroughly addressed in the Mayo data (family-based designs and genomic controls) than in the NINDS dataset.

Third, given the vast number of analyses performed, the threshold for claiming formal statistical significance needs careful consideration. We have used conservative adjustments, but these may be warranted so as to minimize undue emphasis on potentially false-positive results. Nevertheless, a number of genetic variants identified with either of the three strategies as potentially important with unadjusted p-values may warrant further consideration and replication efforts. This may be particularly enticing for the variants proposed with 2 different strategies or even all 3 strategies.

Of the three strategies that we examined, the joint analysis has the best power. This has been demonstrated already by Skol et al. in the setting of comparing two-stage versus joint analyses for genome-wide data for the typical fractions of SNPs being tested in the second stage [26]. The gain in power has always been considered the traditional advantage of meta-analysis in all disciplines where this methodology has been adopted [27], [28]. This is true however primarily when there is no large between-study heterogeneity [27]. At the same time, heterogeneity testing may also give us some useful insights and this may become more important when many datasets are available [29]. In our empirical evaluation, the SNPs that were proposed by each strategy typically had no measurable or minimal between-dataset heterogeneity.

Traditionally, publication bias has been a major threat to the validity of meta-analysis results. The public availability of databases from genome-wide association studies provides an excellent setting where the problem of publication bias can be minimized or even extinguished [8], [9]. This provides an additional argument in favor of making these data-rich experiments publicly available.

Some genetic effects for common variants may be small and readily detectable with genome-wide association studies of very small sample size. Age-related macular degeneration provides one such successful example [30], [31]. However, other genetic variants currently emerging from massive-testing approaches seem to have small or even very small genetic effects [32], [33]. This latter scenario may be far more frequent and even small ORs would still be important to identify for variants that have a considerable frequency in the population. This suggests that there should be an a priori consideration that meta-analysis should be performed on all genome-wide association studies conducted on the same disease. Investigators in the field of type 2 diabetes have already anticipated such a prospective meta-analysis through the IGWANA project [34]. This concept needs to be extended across diverse fields of human genome epidemiology. Meta-analyses may be updated also in a cumulative fashion, when new data appear [35], [36]. Ideally, different teams of investigators should also discuss in advance the plans for a meta-analysis. This may entail agreeing on using common genotyping platforms and/or creating plans for enhancing the consistency of the databases across different studies.
